# Living alone predicts non-home discharge post elective hip arthroplasty: A matched-pair cohort study

**DOI:** 10.1371/journal.pone.0316024

**Published:** 2025-01-02

**Authors:** Benjamin K. Agnor, Ziyad O. Knio, Zhiyi Zuo

**Affiliations:** 1 School of Medicine, University of Virginia, Charlottesville, VA, United States of America; 2 Department of Anesthesiology, University of Virginia Health, Charlottesville, VA, United States of America; South Valley University Faculty of Medicine, EGYPT

## Abstract

The impact of home support and interaction with family members on recovery and perioperative outcomes remains unclear. We determined whether living alone was predictive of discharge disposition following total hip arthroplasty (THA). Data were from American College of Surgeons National Surgical Quality Improvement Program participating hospitals in 2021. The primary endpoint was discharging disposition. A total of 1716 patients living alone and 3961 with others at home were identified. The 1:1 propensity-matched cohort included 3248 total patients (1624 in each group). On univariate analysis, living alone was associated with non-home discharge (22.0% [358/1624] vs. 10.5% [170/1623]; odds ratio [OR], 2.42 [95% CI, 1.98 to 2.94]; *P* < .001), need for services in those returning home (63.1% [799/1266] vs. 57.7% [839/1453]; OR, 1.25 [95% CI, 1.07 to 1.46]; *P* = .004), and increased length of hospital stay (2.05 vs. 1.72 days; mean difference, 0.34 [95% CI, 0.18 to 0.49]; *P* < .001). On multivariable analysis, living alone remained an independent predictor of non-home discharge (adjusted odds ratio, 2.84 [95% CI, 2.30 to 3.54]; c = 0.734). Thus, compared to propensity-matched THA patients with others at home, those living alone experience a much greater rate of non-home discharge and need for support.

## Introduction

Primary total hip arthroplasty (THA) remains the gold standard procedure for end-stage hip osteoarthritis in relieving pain and regaining joint function [1]. The volume of THA procedures is projected to increase due to the higher demand for improved mobility and quality of life in a growing elderly population [2]. In a recent study using data from the Centers of Medicare & Medicaid Services, Shichman et al. (2023) projects that the annual volume of primary THA in the United States will exceed over 700,000 by 2040 and almost 2 million by 2060 [3]. In 2010, the mean age of patients undergoing primary THA was 66, and the prevalence of THA in the United States among adults aged 50 years or older was 2.34% [2].

While THA is among the five most common and fastest-growing procedures in the United States due to its high success rate and cost effectiveness [4], it is crucial to understand trends in complications and associated risk factors to optimize patient outcomes. According to Patel et al. (2023), complications arise in 27.32% of primary THA cases, with postoperative anemia being the most common complication at 25.20% [1]. Other common complications following THA are the development of postoperative delirium (POD) and postoperative cognitive dysfunction (POCD) [5]. Per Kitsis et al. (2022), total hip and knee arthroplasty patients experience POD with a median incidence of 14.8% and POCD with a median incidence of 19.3% at one week and 10% at three months [5]. Patients who suffer from POD or POCD are at risk for poor outcomes including a prolonged hospital stay, increased mortality, and leaving the workforce prematurely [6, 7].

These postoperative complications, along with other patient-related factors, also influence whether a patient is discharged home or to a rehabilitation facility following THA [8–10]. While it is becoming more common to discharge patients directly home following total joint arthroplasty (TJA) [11], evidence-based approach is needed to help identify and risk-stratify home-discharged patients who are at highest risk of postoperative complications. Home conditions, specifically the presence of home caregiver support, may be an important factor in determining discharge location as well as predicting patient outcomes following discharge.

This retrospective study was designed to investigate whether living alone impacted discharge disposition (home versus non-home) following elective THA in a matched cohort sample. The authors hypothesized that living alone would increase non-home discharge.

## Methods

### Study design and data sources

This study was a retrospective cohort analysis of the data from a national de-identified database. Thus, it was exempt from Institutional Review Board approval. The American College of Surgeons National Surgeons Quality Improvement Program (ACS-NSQIP) 2021 database was queried, with THA defined by the Current Procedural Terminology code 27130. The ACS-NSQIP is a nationally validated program that has about 700 voluntarily participating hospitals and whose case selection was performed systematically.

### Patient inclusion and exclusion criteria

The following criteria were used to generate a homogenous study sample. Only elective cases were included. Cases with bone fracture stated in the diagnosis were excluded. The exclusion criteria also included 1) patients admitted to the hospital for longer than one day preceding surgery, 2) patients with preoperative documentation of end-stage renal disease, metastatic disease, sepsis, or bleeding diathesis, and 3) patients with American Society of Anesthesiologist Physical Status (ASA) classification 5 (moribund, not expected to survive without procedure) [12]. There were no modifications made to the ACS-NSQIP classifications for these variables. These inclusion and exclusion criteria were used to generate a sample that could be generalized to the general population of patients for elective THA. These criteria were decided by the researchers *a priori*. All cases received either spinal anesthesia or general anesthesia as the principal anesthetic technique. All cases were required to have a documentation of home support (living alone versus with others at home).

### Measurements and data handling

The primary independent variable was home support (living alone versus with others at home). Other independent variables included age, sex, body mass index (BMI), hypertension, insulin-dependent diabetes, current smoker, chronic obstructive pulmonary disease (COPD), congestive heart failure (CHF), chronic steroid use, functional status, fall within 6 months, dementia, ASA classification, and anesthetic technique (spinal versus general).

The primary endpoint was discharge disposition (home versus non-home). For those discharged home, requiring home services was a major secondary endpoint.

Secondary endpoints also included functional status at discharge, postoperative delirium, hospital length of stay, and 30-day event rates for unplanned resource utilization, wound complications, systemic complications, bleeding requiring transfusion, and mortality. Unplanned resource utilization included unplanned readmission and return to the operating room. Wound complications included superficial surgical site infection (SSI), deep incisional SSI, organ space SSI, and wound dehiscence. Systemic complications included cardiac arrest, myocardial infarction, stroke, reintubation, pneumonia, deep venous thrombosis, pulmonary embolism, bleeding, sepsis, septic shock, and acute kidney injury.

### Statistical analysis

R version 4.3.1 (R Core Team, Vienna, Austria) was used to perform statistical analysis [13]. Patients living alone were identified, and those with others at home were matched using propensity scores computed by the following patient demographics and medical comorbidities: age, sex, BMI, hypertension, diabetes, smoking, COPD, CHF, chronic steroid use, functional status, ASA classification, anesthetic technique, fall history, and dementia. A 1:1 logistic regression covariate estimates with no replacement was used. Cases with missing data were excluded from the analysis in order to promote the creation of balanced cohorts, but with two exceptions: those with unknown functional status were assumed to be independent, and those with unknown fall history were assumed to have not fallen within 6 months given the observed distribution of responses for these two variables. Propensity scoring has been utilized widely to reduce selection bias in cohort studies [14, 15]. Standardized mean differences were used to assess balance among matched pairs, with a standardized mean difference < 0.1 denoting adequate balance [16]. A maximum propensity score difference between groups was not specified *a priori*.

The associations between living alone and various endpoints outcomes were first examined by univariate analyses. Categorical variables were assessed by Pearson’s chi-squared test without continuity correction while hospital length of stay was tested by Student’s t-test. Additionally, chi-square methodology was used to calculate odds ratios (OR), 95% confidence intervals (CI), and fragility indices [17]. The fragility index indicates that changes of how many subject-dependent classifications alter the statistical significance of a hypothesis test. A greater number in the fragility index suggests a more robust difference between groups [18, 19].

A multivariable analysis was performed on the primary outcome. Multiple logistic regression modeling was used and adjustments were made for age, sex, BMI, hypertension, diabetes, smoking, COPD, CHF, chronic steroid use, functional status, fall history, dementia, ASA classification, anesthetic technique, and home support when univariate testing showed significance at α ≤ 0.05. The selection of these variables was decided *a priori* and based on the availability of the data for analysis. As with matching, only complete cases were analyzed. Missing data were not imputed. Backwards stepwise model adjusted by Akaike information criterion was used to determine the subsequent variable selection. Results of independent predictors were presented as adjusted odds ratios (AOR) and 95% confidence interval (CI), and the c-statistic was used to assess model discrimination [20].

Continuous variables and categorical variables were presented as mean (standard deviation) and frequency (%), respectively. All hypothesis tests were two-sided, with α ≤ 0.05 as having a significant difference.

## Results

**Fig 1:**
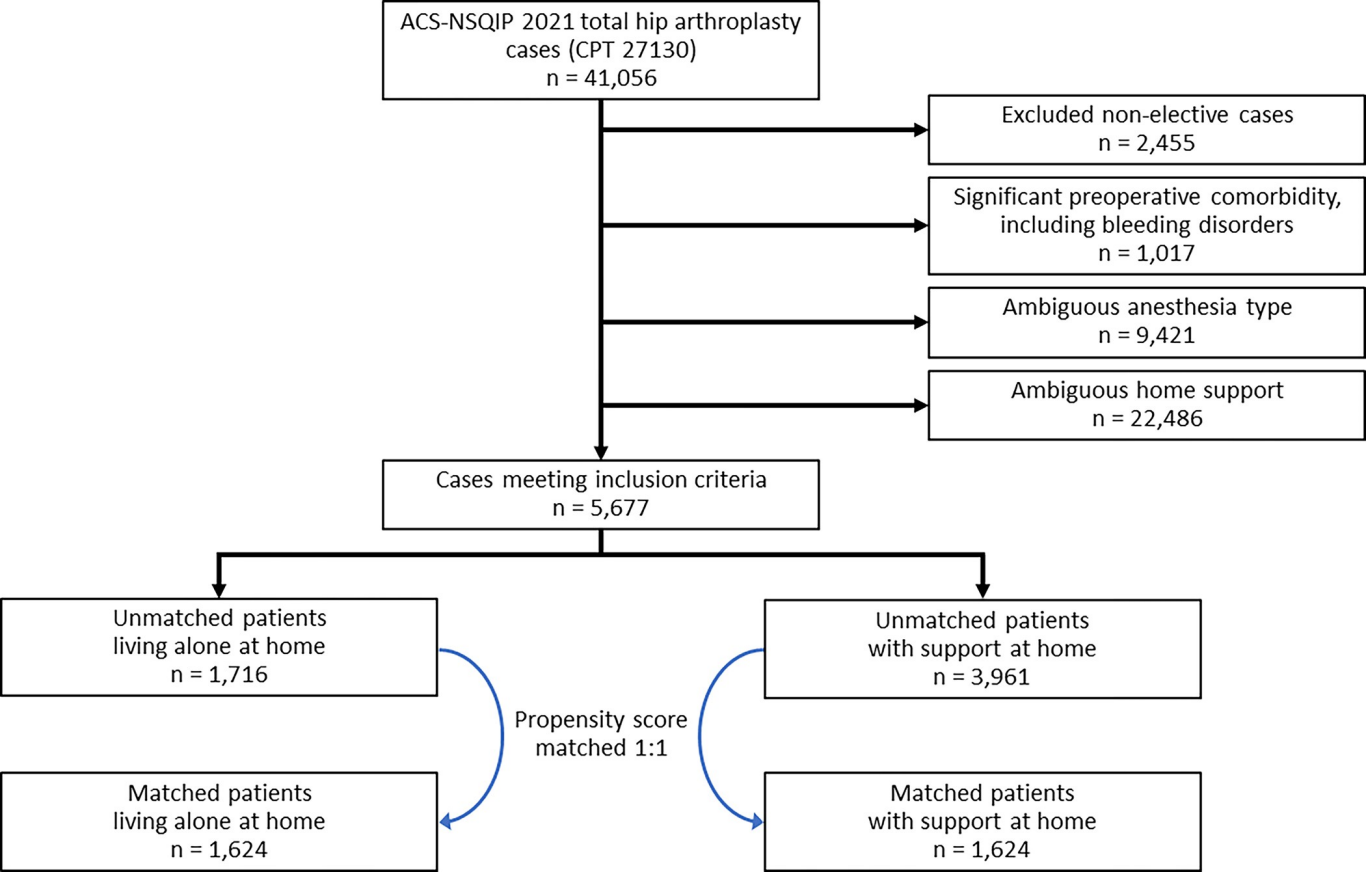
Consolidated standards of reporting trials diagram detailing selection of patients within each cohort, including numbers of patients in each cohort before and after matching. Abbreviations: ACS-NSQIP, American College of Surgeons National Surgical Quality Improvement Program.

**Fig 2:**
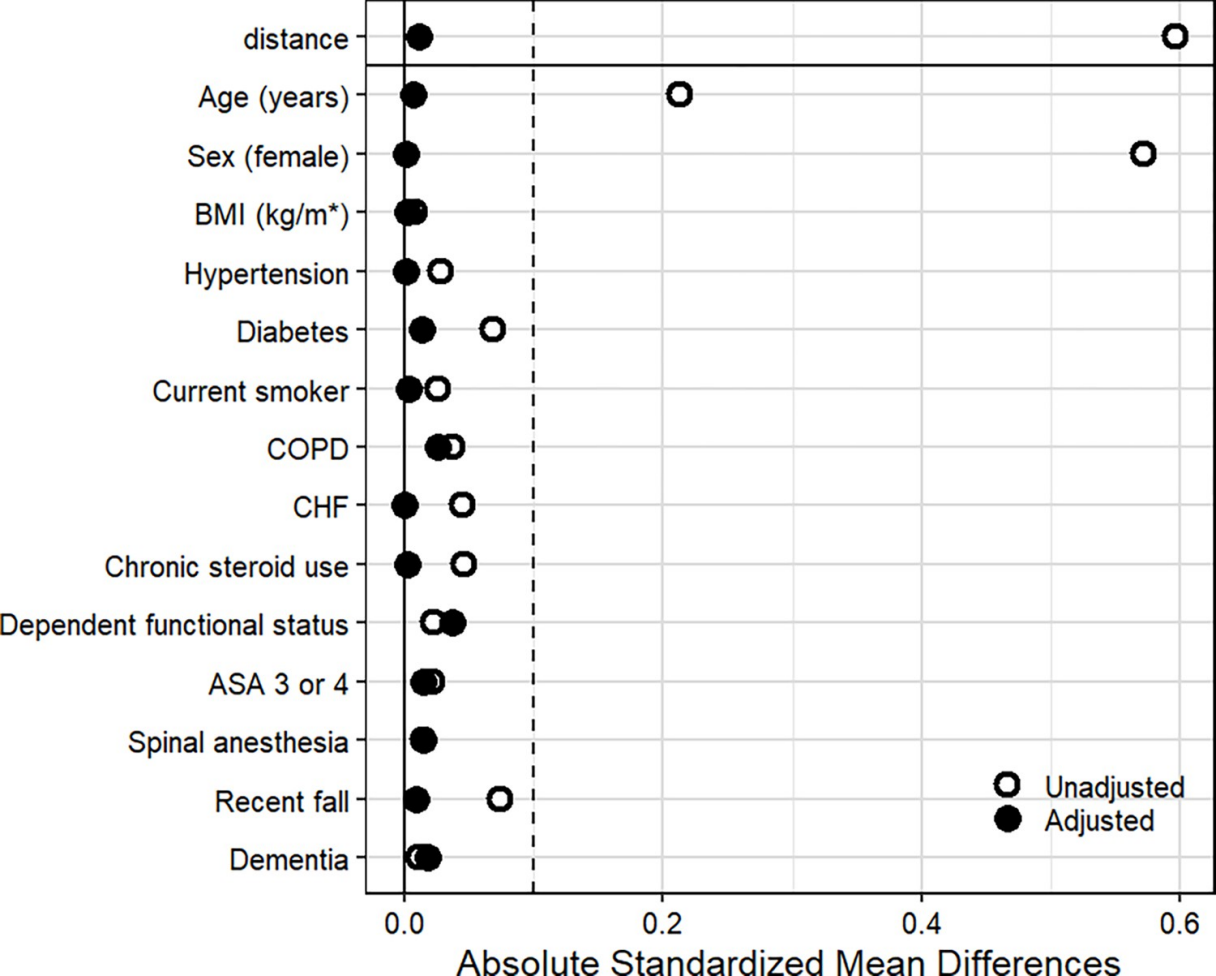
Absolute standardized mean differences before (n = 5677) and after (n = 3248) matching those living alone to those living with others at home 1:1 by propensity score. Abbreviations: ASA, American Society of Anesthesiologists; BMI, body mass index; CHF, congestive heart failure; COPD, chronic obstructive pulmonary disease.

Of 5677 THA patients that met inclusion criteria, 1716 (30.2%) were living alone at the time of surgery. After 1:1 propensity score matching of patient demographics and medical comorbidities, a total of 3248 patients were identified (1624 in each study group) (Fig 1). Balance between cohorts was evidenced by standardized mean differences < 0.1 (Fig 2). For the sake of matching, those with unknown functional status were assumed to be independent given that this was the case for 96.7% of subjects with a functional status response. For the sake of matching, those with unknown fall history were assumed to have not fallen within 6 months given that this was the case for 93.8% of subjects with a fall history response. The patient demographics are subsequently summarized without making such an assumption, reporting only on those with valid responses for functional status and fall history. The distribution of patient demographics and medical comorbidities in those living alone versus with others at home both before and after matching are presented in Table 1.

**Table 1:** Demographics and medical comorbidities by home living conditions, before and after propensity score matching.

	Before propensity score matching			After propensity score matching		
	Number/total (%)			Nnumber/total (%)		
Characteristic	With others at home (n = 3961)	Living alone (n = 1716)	*P* value	With others at home (n = 1624)	Living alone (n = 1624)	*P* value
Age, mean (SD), years	79.3 (3.6)	80.1 (3.8)	< .001	80.1 (3.8)	80.1 (3.8)	.854
Female sex	2191/3961 (55.3%)	1348/1716 (78.6%)	< .001	1277/1624 (78.6%)	1278/1624 (78.7%)	.966
BMI, mean (SD), kg/m^2^	28.6 (5.3)	28.5 (5.6)	.586	28.7 (5.5)	28.6 (5.6)	.955
Hypertension	2692/3961 (68.0%)	1153/1716 (67.2%)	.568	1086/1624 (66.9%)	1085/1624 (66.8%)	.970
Insulin-dependent diabetes	105/3961 (2.7%)	29/1716 (1.7%)	.029	32/1624 (2.0%)	29/1624 (1.8%)	.698
Current smoker	128/3961 (3.2%)	64/1716 (3.7%)	.340	62/1624 (3.8%)	61/1624 (3.8%)	.927
COPD history	207/3961 (5.2%)	102/1716 (5.9%)	.273	109/1624 (6.7%)	99/1624 (6.1%)	.474
CHF history	142/3961 (3.6%)	75/1716 (4.4%)	.156	71/1624 (4.4%)	71/1624 (4.4%)	>.999
Chronic steroid use	143/3961 (3.6%)	79/1716 (4.6%)	.076	74/1624 (4.6%)	75/1624 (4.6%)	.933
Dependent functional status	135/3952 (3.4%)	54/1713 (3.2%)	.612	56/1622 (3.5%)	46/1621 (2.8%)	.316
Recent fall	210/3714 (5.7%)	117/1579 (7.4%)	.015	111/1527 (7.3%)	115/1496 (7.7%)	.662
Dementia	130/3961 (3.3%)	52/1716 (3.0%)	.621	44/1624 (2.7%)	49/1624 (3.0%)	.599
ASA 3 or 4	2379/3955 (60.2%)	1012/1710 (59.2%)	.494	966/1624 (59.5%)	954/1624 (58.7%)	.668
Spinal anesthesia	2125/3961 (53.6%)	936/1716 (54.5%)	.533	868/1624 (53.4%)	880/1624 (54.2%)	.673

Abbreviations: ASA, American Society of Anesthesiologists; BMI, body mass index; CHF, congestive heart failure; COPD, chronic obstructive lung disease.

On univariate analysis, living alone was associated with non-home discharge (22.0% [358/1624] vs. 10.5% [170/1623]; OR, 2.42 [95% CI, 1.98 to 2.94]; *P* < .001), need for services in those returning home (63.1% [799/1266] vs. 57.7% [839/1453]; OR, 1.25 [95% CI, 1.07 to 1.46]; *P* = .004), and increased length of stay (2.05 vs. 1.72 days; mean difference, 0.34 [95% CI, 0.18 to 0.49]; *P* < .001). The fragility index for non-home discharge was robust (124). Living alone was not associated with functional status at discharge, postoperative delirium, or rates of unplanned resource utilization, wound complications, systemic complications, bleeding, or mortality (Table 2).

**Table 2:** Univariate analyses by home living conditions (living alone versus with others).

	Number/total (%)				
Outcome	With others at home (n = 1624)	Living alone (n = 1624)	Odds ratio (95% CI)	*P* value	Fragility index
**Non-home discharge**	170/1623 (10.5%)	358/1624 (22.0%)	2.42 [1.98 to 2.94]	< .001	142
**Discharge home with services**	839/1453 (57.7%)	799/1266 (63.1%)	1.25 [1.07 to 1.46]	.004	21
**Dependent functional status at discharge**	661/1574 (42.0%)	665/1581 (42.1%)	1.00 [0.87 to 1.16]	.970	54
**Delirium**	24/692 (3.5%)	39/701 (5.6%)	1.64 [0.98 to 2.76]	.060	2
**Length of stay, mean (SD), days**	1.72 (1.97)	2.05 (2.42)	0.34 [0.18 to 0.49]	< .001	-
**Unplanned resource utilization (composite)**	81/1624 (5.0%)	96/1624 (5.9%)	1.20 [0.88 to 1.62]	.246	11
Unplanned readmission	71/1624 (4.4%)	89/1624 (5.5%)	1.27 [0.92 to 1.75]	.144	7
Unplanned reoperation	32/1624 (2.0%)	39/1624 (2.4%)	1.22 [0.76 to 1.96]	.401	10
**Wound complications (composite)**	32/1624 (2.0%)	26/1624 (1.6%)	0.81 [0.48 to 1.36]	.427	9
Superficial SSI	20/1624 (1.2%)	14/1624 (0.9%)	0.70 [0.35 to 1.39]	.301	6
Deep incisional SSI	2/1624 (0.1%)	5/1624 (0.3%)	2.50 [0.49 to 12.93]	.256	3
Organ space SSI	8/1624 (0.5%)	6/1624 (0.4%)	0.75 [0.26 to 2.16]	.592	5
Wound dehiscence	3/1624 (0.2%)	6/1624 (0.4%)	2.00 [0.50 to 8.03]	.317	3
**Systemic complications (composite)**	28/1624 (1.7%)	37/1624 (2.3%)	1.33 [0.81 to 2.18]	.259	7
Cardiac arrest	2/1624 (0.1%)	3/1624 (0.2%)	1.50 [0.25 to 8.99]	.654	5
Myocardial infarction	5/1624 (0.3%)	7/1624 (0.4%)	1.40 [0.44 to 4.43]	.563	5
Stroke	1/1624 (0.1%)	5/1624 (0.3%)	5.01 [0.58 to 42.95]	.102	2
Unplanned reintubation	4/1624 (0.2%)	4/1624 (0.2%)	1.00 [0.25 to 4.01]	>.999	6
Pneumonia	10/1624 (0.6%)	12/1624 (0.7%)	1.20 [0.52 to 2.79]	.669	7
Deep venous thrombosis	6/1624 (0.4%)	10/1624 (0.6%)	1.67 [0.61 to 4.61]	.316	4
Pulmonary embolism	5/1624 (0.3%)	7/1624 (0.4%)	1.40 [0.44 to 4.43]	.563	5
Sepsis	2/1624 (0.1%)	5/1624 (0.3%)	2.50 [0.49 to 12.93]	.256	3
Septic shock	5/1624 (0.3%)	2/1624 (0.1%)	0.40 [0.08 to 2.06]	.256	3
Acute kidney injury	0/1624 (0.0%)	2/1624 (0.1%)	-	.157	4
**Bleeding**	49/1624 (3.0%)	49/1624 (3.0%)	1.00 [0.67 to 1.49]	>.999	19
**Mortality**	5/1624 (0.3%)	5/1624 (0.3%)	1.00 [0.29 to 3.46]	>.999	6

Abbreviations: CI, confidence interval; SSI, surgical site infection.

On multivariable analysis, non-home discharge was independently predicted by age (AOR, 1.10 [95% CI, 1.07 to 1.13]; *P* < .001), BMI (AOR, 1.03 [95% CI, 1.01 to 1.05]; *P* < .001), hypertension (AOR, 1.21 [95% CI, 0.95 to 1.54]; *P* = .120), dependent functional status (AOR, 3.32 [95% CI, 2.06 to 5.30]; *P* < .001), fall within 6 months (AOR, 1.72 [95% CI, 1.22 to 2.40]; *P* = .001), dementia (AOR, 2.18 [95% CI, 1.29 to 3.62]; *P* = .003), ASA 3 or 4 classification (AOR, 1.97 [95% CI, 1.56 to 2.50]; *P* < .001), general anesthesia (AOR, 1.77 [95% CI, 1.44 to 2.18]; *P* < .001), and living alone (AOR, 2.84 [95% CI, 2.30 to 3.54]; *P* < .001). The model demonstrated good discrimination (model c-statistic, 0.734) and calibration (Hosmer-Lemeshow *P* = 0.416).

## Discussion

This study determined that for patients undergoing primary THA, living alone was predictive of non-home discharge and increased hospital length of stay. Patients who were living alone were also more likely to require home services if discharged to home. In our study, patients living alone experienced no appreciable difference in functional status at discharge, postoperative delirium, rates of unplanned readmission or reoperation, wound or systemic complications, bleeding, or mortality when compared to patients with home support.

To our knowledge, only one other study has specifically investigated the impact of living alone on postoperative outcomes [21]. In that study, Fleischman et al. (2018) prospectively assessed the safety and efficacy of direct home discharge for patients living alone [21]. However, their study included both primary total hip and knee arthroplasty patients from a single institution with direct home discharge as the standard of care regardless of home support status. Conversely, our study focused solely on primary THA using data from the ACS-NSQIP, a surgical outcomes database contributed by hundreds of institutions throughout the United States. Despite the differences in study design, conclusions were concordant, with both the present study and that of Fleischman et al. (2018) demonstrating that living alone was associated with prolonged hospital stays and increased utilization of home health services.

### Statistics on postoperative home support

To date, there is a paucity of evidence to help determine what percentage of patients receiving primary THA are living independently or with home support. Based on 2021 data from the ACS-NSQIP, approximately 30% of patients receiving primary THA with reported home support status were found to be living alone. However, this metric may not be valid as over half of these cases did not report home support status. Studies by Iwata et al. (2023) and Fleischman et al. (2018) showed approximately 20% of their study participants undergoing primary THA were reported to be living alone, however these metrics were collected from single-institution databases and may not be generalizable to larger populations [10, 21].

### Social factors affecting patient outcomes

Given that a significant number of patients receiving primary THA are living independently, there has been much research aimed at determining whether living and social conditions, such as caregiver support at home, affect patient outcomes postoperatively. Fleischman et al. (2018) showed no increase in complications or unplanned clinical events for patients living alone compared to those living with others. Additionally, there were no significant differences in functional outcomes or pain relief in patients living alone or living with others [21].

However, prior systematic reviews have examined the impact of social determinants on patient-reported outcomes and adverse events following TJA [22, 23]. Karimi et al. (2023) found that patients with more social deprivation had a higher proportion of non-home discharge and lower improvements from baseline patient-recorded outcome measures following TJA [22]. A systematic review and meta-analysis by Wylde et al. (2019) showed evidence that social support could be a prognostic factor for some patient-reported outcome measures following total joint replacement [23]. Notably, both reviews reported appreciable limitations in their findings due to the methodological quality of available studies and to the inconsistent measurement of social support or deprivation. To combat the complex and multidimensional nature of social support, future studies must specify the social factors they are measuring and utilize metrics that effectively capture social determinants.

### Factors affecting discharge location

Numerous retrospective studies have investigated predictors of discharge destination following TJA [8–10, 24, 25]. Additionally, multiple authors are credited with developing and validating pre-operative risk assessment tools to predict discharge destination for patients undergoing TJA [26–29]. Interestingly, two out of four known discharge prediction tools utilize home caregiver status in their scoring [27, 28]. While our study focused solely on living alone as a predictive factor of discharge destination, other studies identified multiple predictors for non-home discharge including older age [8–10, 24, 25], female gender [9, 30], certain comorbidities including obesity [8, 30] and pulmonary disease [9, 30], postoperative functional status [8, 30], and even patient expectation of discharge destination [25]. The studies that included living alone in their analysis had findings consistent with our study [8, 10, 25].

### Factors affecting hospital length of stay

Similarly to discharge destination, prior studies also have looked for factors that influence hospital length of stay after TJA [9, 21, 24, 31]. Correspondingly, Fleischman et al. (2018) determined that living alone was associated with longer inpatient stays [21]. Other retrospective studies found strong associations linking length of stay with age [24], diabetes [9], pre-existing cognitive impairment [31], discharge destination [9], and even day of surgery [9]. These results suggest that a combination of patient and organizational factors play as drivers in determining hospital length of stay.

### Implications of our findings

Our findings suggest that patients living alone may require additional support postoperatively as well as longer inpatient stays to receive sufficient patient education and coordinate support services at home. Patients living alone may lack the physical, cognitive, and emotional support and interactions needed in the initial recovery phase following THA, necessitating a discharge destination that provides ongoing caregiver support. Fortunately, there is no evidence to date associating lack of home support with negative postoperative outcomes following THA. However, animals studies have shown that familial support reduces POCD, possibly via inhibition of neuroinflammation and activation of the lateral habenula-ventral tegmental area neural circuit after surgery [32–34]. These novel findings from animal studies suggest that the presence of home support may influence postoperative neuropsychological outcomes, however there is a need for clinical studies to better understand these phenomena and their implication in patients living alone where social interactions are very limited. In this context, it is worth noting that there was a trend for living alone patients to have a higher incidence of POD (OR: 1.64 [0.98 to 2.76], P = 0.06). Since the diagnosis of POCD requires neurocognitive assessment before and after surgery [6], there is no data on the incidence of POCD in this retrospective analysis.

Given that patients undergoing THA have unique medical and social backgrounds and require varying levels of support in the postoperative period, it is crucial that healthcare providers risk-stratify patients early in the preoperative phase to ensure proper discharge planning and deliver the optimal level of care throughout recovery and rehabilitation. Our study emphasizes the importance of including home support in these risk assessments to obtain a more comprehensive view of our patients’ postoperative trajectories.

### Strengths

The study is strengthened by reporting of fragility indices for all outcomes, complementing the *P* value with an interpretable measure of patient classification on statistical significance [18, 19]. At a minimum, the classification of 142 discharge dispositions would need to be changed to the opposite class to alter the statistical significance of these findings. This study is further strengthened by propensity score matching those living alone to those with others at home in order to control for selection bias [16]. The cohort living alone before propensity score matching had an overrepresentation of elderly patients, females, non-diabetics, and recent falls. Matching produced balanced cohorts, as evidenced by post-matching univariate analyses (all *P* > .05) and a marked reduction in absolute standardized mean differences.

### Limitations

There are several important limitations that are inherent to the study design. This was an observational study, and as such the defined endpoints and covariates were limited to those captured by the ACS-NSQIP database. Home support was not documented in 22486 of 28163 patients otherwise meeting inclusion criteria, reducing the power of this study and potentially introducing selection bias. This supports the use of propensity score matching as one method to reduce sampling bias, though matching is not without limitations [35]. Additionally, the characters of individuals living with the supported patients are not reported in the database; factors like age, functional status, health literacy, and chronic illness are likely influential but to an unknown degree. The screening test for postoperative delirium is also not documented. Thus, it is a distinct possibility that the screening methodology has not been validated for the 93 patients with dementia (2.9% of the matched sample). The study design also did not allow for exclusion or stratification by intraoperative factors, such as transfusion requirements. This study design was used to allow the application of our findings to general population of patients with elective hip arthroplasty. Instead, some perioperative complications, such as transfusion requirements, are studied as secondary endpoints in the analysis. Lastly, the results of this study cannot be generalized to patients meeting the *a priori* exclusion criteria.

## Conclusions

In summary, the present study demonstrates the unique challenges faced by patients living alone following THA. These patients are more likely to have longer hospital stays and require discharge to non-home rehabilitation facilities, emphasizing the need for comprehensive risk assessment and discharge planning to ensure adequate patient education, support and coordination of postoperative care. Future studies should consider including a wider range of surgical procedures for generalization of findings, further investigating the effects of home support and social interaction on postoperative outcomes including POD and POCD, and further characterizing the mechanisms behind living alone and its associations with non-home discharge and prolonged hospital length of stay. These studies may ultimately identify approaches to better prepare patients who live alone for excellent recovery after surgery.
